# Supplementary material for: Reconfigurable all-dielectric metalens with diffraction-limited performance
Source: Nat Commun. 2021 Feb 22;12:1225. doi: 10.1038/s41467-021-21440-9 (PMC7900249; doi:10.1038/s41467-021-21440-9)
Supplement: Supplementary file 1 — Supplementary Information [file 41467_2021_21440_MOESM1_ESM.pdf]

Supplementary Information for

"Reconfigurable All-dielectric Metalens with Diffraction Limited Performance"

Shalaginov, An, et al.

Supplementary Note 1 – Transmission properties of meta-atoms under different states

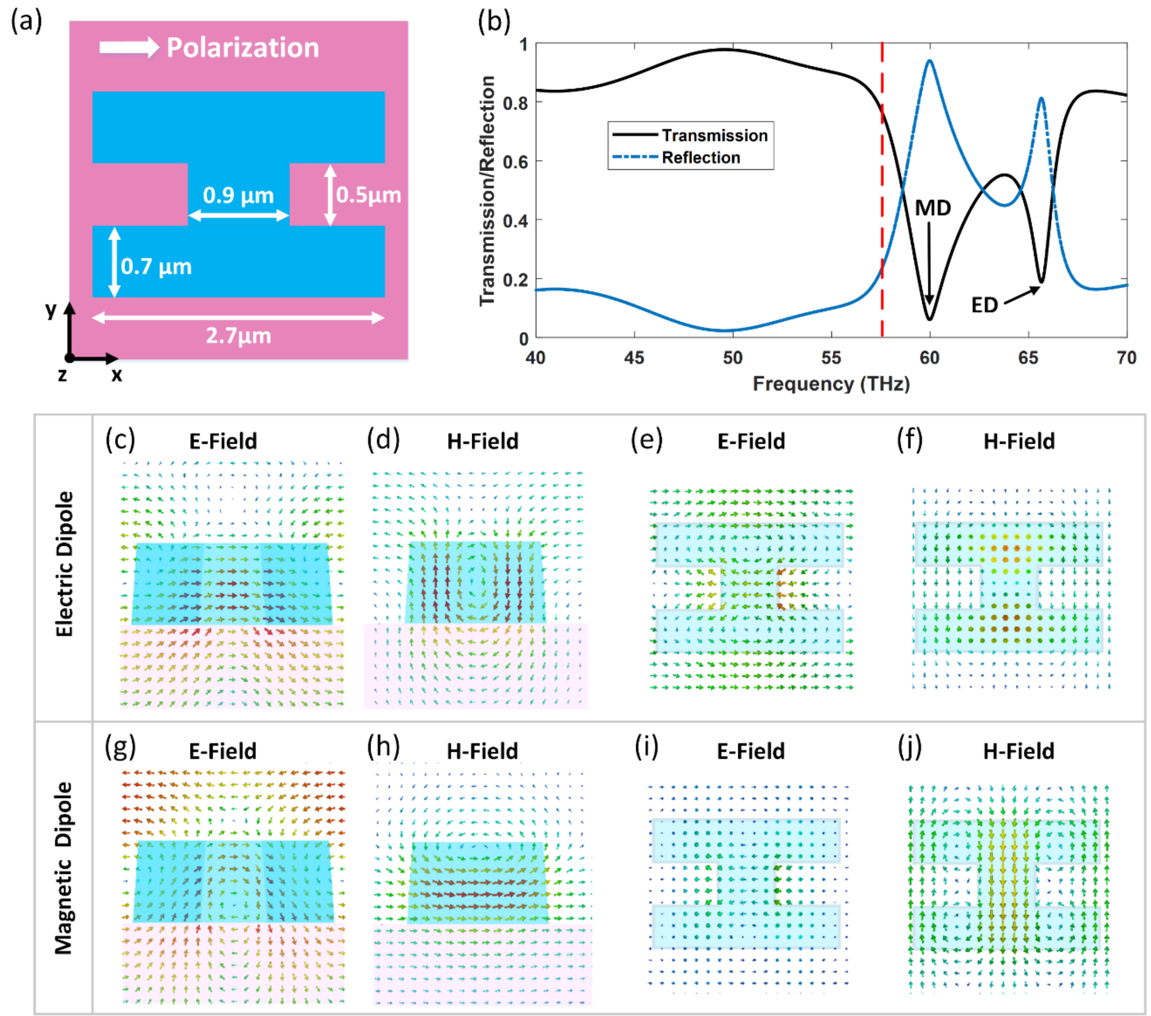

Supplementary Fig. 1. (a) Schematic top-view of an H-shaped GSST-on-CaF2 meta-atom in the amorphous state; (b) transmission and reflection spectra of an infinite array formed by a periodic arrangement of the meta-atom in (a), showing two spectrally separated resonances (MD and ED); The operating frequency (57.7 THz) is marked in red dashed lines. (c-f) field distributions for the ED resonance at different planes: (c) x-z plane; (d) y-z plane; (e-f) x-y planes; (g-j) field distributions for the MD resonance at different cutting planes: (g) x-z plane; (h) y-z plane; (i j) x-y planes.

A Huygens type metasurface is able to excite electric dipole (ED) and magnetic dipole (MD) resonances within its fundamental building blocks - meta-atoms. Here, we take an H-shaped GSST-on-CaF_2 meta-atom as an example and analyze its electric and magnetic resonant behavior under both amorphous (Supplementary Fig. 1) and crystalline (Supplementary Fig. 2) states, as well as the methods used to engineer optical transmission properties (phase and amplitude) via tuning the ED and MD resonances.

Supplementary Figure 1 illustrates the transmission (reflection) properties of an infinite array composed of H-shaped GSST meta-atoms (blue region in Supplementary Fig. 1a) sitting on top of an infinite CaF_2 substrate (pink region in Supplementary Fig. 1a). The simulation was performed using the full wave simulation tool CST Microwave Studio, with a linear-polarized incident plane wave that propagates perpendicular to the GSST-CaF_2 interface and illuminates from the substrate side. The transmission and reflection spectral responses shown in Supplementary Fig. 1b indicate two distinct dipole resonances at 59.98 THz and 65.65 THz, respectively, corresponding to the MD and ED resonances which are the two lowest existing multi-polar resonances. Supplementary Figs. 1c-j show the electric and magnetic field distributions on different cutting planes at the MD and ED resonant frequencies. The electric dipole shows an E-field concentrated at the center of the meta-atom (Supplementary Fig. 1c) and a vortex-like H-field (Supplementary Fig. 1d) surrounding the E-field. Correspondingly, Supplementary Figs. 1g-h clearly show the magnetic resonance behavior with the H-field concentrated at the center of the meta-atom (Supplementary Fig. 1h) and a vortex-like E-field (Supplementary Fig. 1g) surrounding the H-field.

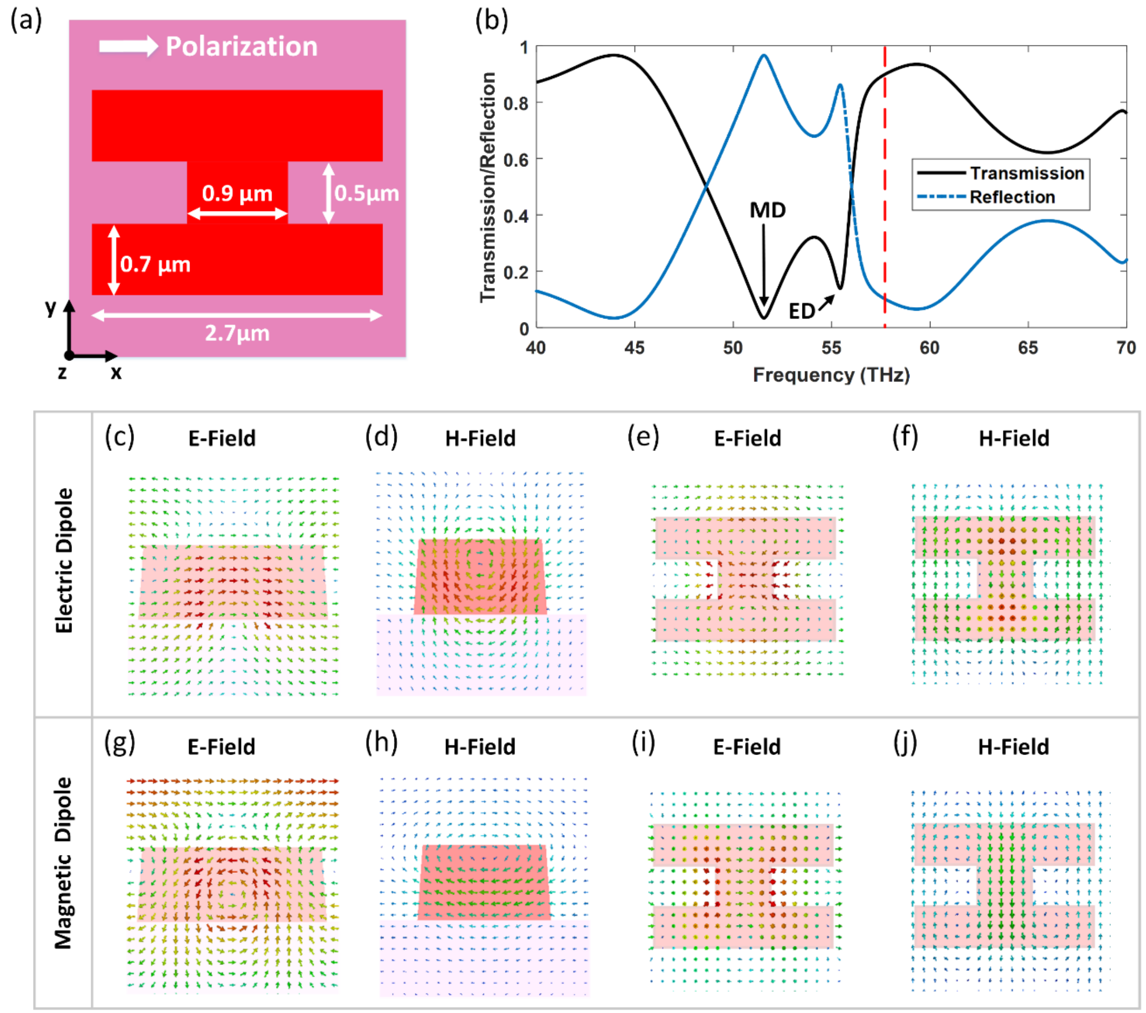

Supplementary Fig. 2. (a) Schematic top-view of an H-shaped GSST-on-CaF_2 meta-atom in the crystalline state; (b) transmission and reflection spectra of an infinite array formed by a

periodic arrangement of the meta-atom in (a), showing two spectrally separated resonances (MD and ED); The operating frequency (57.7 THz) is marked in red dashed lines. (c-f) field distributions for the ED resonance at different planes: (c) x-z plane; (d) y-z plane; (e-f) x-y planes; (g-j) field distributions for the MD resonance at different cutting planes: (g) x-z plane; (h) y-z plane; (i-j) x-y planes.

It's worth mentioning that the electric and magnetic resonances can be easily identified since they are well-separated spectrally in Supplementary Fig. 1. These two resonances shift (at different places) when the meta-atom material, GSST, is reconfigured from one state to another. As shown in Supplementary Fig. 2, the ED and MD resonances are red-shifted towards longer wavelengths when GSST is switched from amorphous state to crystalline state (i.e. its refractive index increases). As a result, when the GSST-based meta-atom shown in Supplementary Figs. 1 and 2 is switched from amorphous state to crystalline state, the two dipole resonances are now located at 51.55 THz and 55.45 THz, respectively. The unique ability to tune the MD and ED resonances through controlling the refractive index permits us to independently engineer the meta-atom's transmission properties (phase and amplitude) under different states and thus enables multi-functional metasurface designs.

Supplementary Note 2 – 4-level reconfigurable meta-atom designs

To enable the desired functionalities, the meta-atoms have to be carefully engineered to realize arbitrary phase profiles in both amorphous and crystalline states. Ideally, we should have an infinite number of highly transparent meta-atom structures to fulfil perfectly accurate phase-map targets under both states, however, this is obviously impractical (if not impossible). Alternatively, we have discretized the full phase coverage range into 90-degree intervals (4 steps) for each state, so that every point along an arbitrary phase mask will be mapped to one of these four steps. For arbitrary reconfigurability, it is required that for each step in one state, there must be a structure that can assume each of the four steps in the other state. This requires a total of 16 unique structures, which we refer to as a 4-level or "2-bit" design. We found an optimal set of meta-atom structures with a lattice constant of 3 μm, and thickness of 1.1 μm/1.065μm for the amorphous/crystalline state, so that ED and MD can co-exist in each meta-atom under both states. We conducted a thorough parameter sweep, considering three types of resonators including H-shaped (Supplementary Fig. 3a), I-shaped (Supplementary Fig. 3b) and cross-shaped structures (Supplementary Fig. 3c). A sidewall angle of 85 degrees and 3.2% thickness shrinkage (when reconfigured from amorphous to crystalline state) were applied to the meta-atom models during simulation to account for the impact of fabrication and annealing processes. As shown in Supplementary Figs. 3a-c, each resonator was modeled using four distinct parameters with CST Microwave Studio. For each single meta-atom, unit cell boundary conditions were employed for the calculation of transmission amplitude and phase. Open boundaries are applied in both the positive and negative *z* directions. *x*-polarized incident waves are illuminated from the substrate side and propagate in the *z* direction. Simulated transmission amplitudes and phases of meta-atoms with different shapes are plotted in Supplementary Figs. 3c-d. Through the combination of these different meta-atom structures, we are able to realize relatively high transmission within the entire 0-2π phase range under both amorphous state and crystalline state.

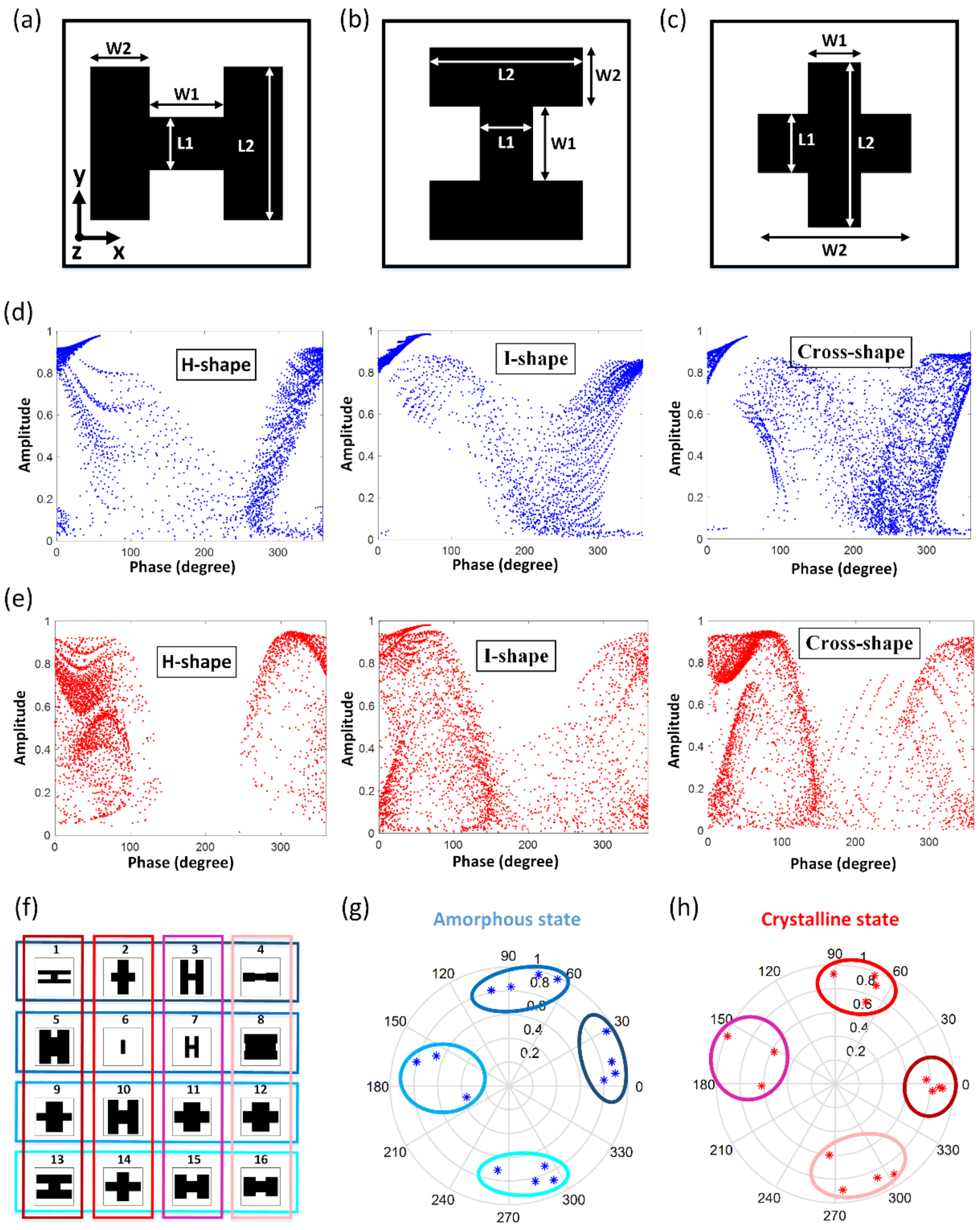

Supplementary Fig. 3. Schematic top-view of an (a) H-shaped, (b) I-shaped and (c) Cross-shaped meta-atom design, with x-polarized incidence; (d-e) scatter diagrams of the transmission phase and amplitude derived with the different shaped meta-atoms shown in (a-c), under amorphous state (blue dots, figure (d)) and crystalline state (red dots, figure (e)), respectively. (f) Schematic top-view of all selected 2-bit meta-atom designs; (g) simulated phase and amplitude of the 16 meta-atoms in amorphous state; (h) simulated phase and amplitude of the 16 meta-atoms in crystalline state.

Sets of 16 meta-atoms (i.e. the 2-bit set shown in Supplementary Fig. 3f) that provide full 2π phase coverage in both states are identified from the simulation results plotted in Supplementary Figs. 3d-e. These sets can be used in designs to generate arbitrary wavefronts under both amorphous and crystalline states. For each discrete phase under one state, four discrete phases covering 2π with about 90° phase intervals could be found at another state. More specifically, in Supplementary Fig. 3f, phase responses of meta-atoms that are circled in red (e.g. meta-atoms or cells #1, 5, 9 and 13) are similar under crystalline state, but are evenly distributed in the 2π range with a 90° interval under amorphous state. Meta-atoms that are circled in blue (e.g. meta-atoms #13, 14, 15 and 16) are similar under amorphous states and different under crystalline state. Complete dimensions of these 16 selected meta-atoms are listed in Supplementary Table 1. All of the listed dimensions refer to the bottom surface of the GSST meta-atoms. The dimensions at the top surface are slightly smaller due to the meta-atom sidewalls slanted at 85°. Phase-delays and transmittance associated with the selected meta-atoms are provided in Supplementary Table **2.**

Supplementary Table 1. Dimensions of meta-atoms used in the meta-optics devices. All lengths and widths in microns, schematic top-views of meta-atoms are shown in Supplementary Fig. 3a-c.

meta-atom number (shape)	1 (I)	2 (+)	3 (H)	4 (H)	5 (H)	6 (I)	7 (H)	8 (I)
L_1, μm	0.5	0.7	0.5	0.5	1.5	0.3	0.3	2.3
W_1, μm	0.4	0.8	0.6	0.9	0.5	0.3	0.4	0.6
L_2, μm	2.5	2.7	2.7	0.7	2.7	0.3	1.7	2.5
W_2, μm	0.3	1.8	0.6	0.9	0.9	0.4	0.3	0.7
meta-atom number (shape)	9 (+)	10 (H)	11 (+)	12 (+)	13 (I)	14 (+)	15 (H)	16 (H)
L_1, μm	0.6	1.1	0.9	0.9	0.9	0.7	0.9	0.9
W_1, μm	1.3	0.7	1.3	1.3	0.5	0.9	0.8	0.8
L_2, μm	2.4	2.7	2.3	2.3	2.7	2.3	1.9	1.5
W_2, μm	2.7	0.8	2.7	2.7	0.7	2.7	0.9	0.9

To further explore and demonstrate the behaviors of the designed reconfigurable meta-atoms, we take a closer look at meta-atoms #13-16 in a wider spectrum range. As shown in Supplementary Fig. 4, when these four meta-atoms are in the amorphous state (with a relatively low refractive index), the operating frequency (57.7 THz, marked in dashed lines) was lower than the MDs or EDs for all four meta-atoms, which resulted in relatively high transmission and similar phase shift. When these meta-atoms are annealed and reconfigured to the crystalline state, the transmission spectral responses of them are red-shifted differently (varied depending on meta-atoms'

dimensions). As a result, these four meta-atoms can support combinations of different modes, leading to different phase delays.

Supplementary Table 2. Phase delays and transmittance of the selected meta-atoms in amorphous and crystalline states of GSST.

cell	amorphous		crystalline	
	phase, °	T, %	phase, °	T, %
1	29.2	88.1	357.6	81.8
2	6.7	81.0	90.8	85.2
3	13.2	78.8	152.4	33.4
4	3.4	64.0	274.1	80.0
5	100.5	66.8	355.8	67.2
6	75.1	93.5	70.0	93.6
7	65.6	97.0	56.1	97.7
8	88.8	69.6	294.2	74.9
9	165.5	64.6	2.6	58.8
10	195.0	13.5	69.6	53.9
11	157.6	43.7	323.1	82.2
12	157.6	43.7	323.1	82.2
13	294.0	54.6	358.1	76.4
14	295.4	77.1	67.4	79.9
15	262.4	51.3	211.6	38.2
16	285.5	69.5	265.3	36.1

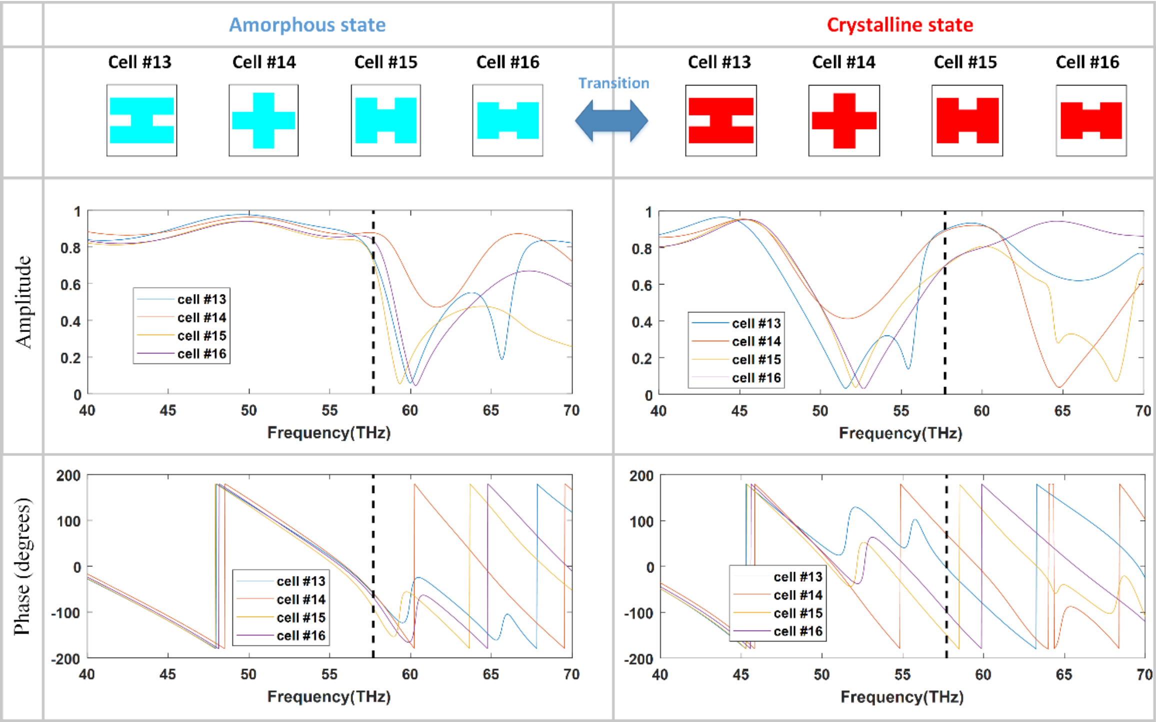

Supplementary Fig. 4. Simulated transmission and phase spectra of meta-atoms #13-16 under amorphous (left) and crystalline (right) state.

Supplementary Note 3 – Figure-of-merit for evaluating and selecting active meta-atoms

During the metasurface design process, we adopted a performance figure-of-merit (FOM) for meta-atom evaluation and optimization prior to full-scale meta-optical system simulation. Within the regime of scalar diffraction theory, the transmittance function of a diffractive optical element (DOE) or a metasurface in our case, can be considered as the summations of the transmittances of all unit cells (i.e., meta-atoms). The far-field amplitude of such structures can be then obtained from the Fourier transformation of the transmittance function, similar to multi-level DOEs[1]. Here, we use a subwavelength grating structure to study the dependence of phase errors on diffraction efficiency. It has been shown that the diffraction efficiency of an arbitrary DOE is directly related to that of a grating[2]. It should be noted that the scalar theory based on diffraction efficiency calculation method has a limited accuracy as the size of the periods becomes comparable to the wavelength. However, this method allows closed-form analytical expressions of the diffraction efficiency without time-consuming numerical modeling, and thus is particularly useful and efficient for evaluating and comparing single meta-atoms or meta-atom groups. After meta-atoms with high FOMs were selected, we employed rigorous Kirchhoff diffraction integration method incorporating the generated phase and amplitude masks to further validate the performance of the entire metasurface.

The transmittance of a grating structure with a continuous phase profile (Supplementary Fig. 5a) along the modulation direction (x-axis) can be described as:

$$t(x) = \sum_{m=-\infty}^{\infty} \delta(x - mT) * rect(x/T) exp(i\phi(x)) , \tag{1}$$

$$\phi(x) = \phi_{\max} x/T , \tag{2}$$

where m represents the m-th diffraction order, T is the grating period, $\phi_{\max}$ is the maximum phase delay within one period and * denotes a convolution operation. The Fourier transform of the transmittance results in its far-field amplitude distribution:

$$F(f) = \sum_{m=-\infty}^{\infty} \delta\left(f - m/T\right) \frac{\sin\left(\pi T\left(\phi_{\max}/2\pi T - f\right)\right)}{\pi T\left(\phi_{\max}/2\pi T - f\right)} , \tag{3}$$

$$f = \sin(\theta)/\lambda_0 , \tag{4}$$

where λ_0 is the wavelength in free space and θ is the first-order diffraction angle. The diffraction efficiency of the m-th order is thus the amplitude value squared:

$$\eta_m = \left[\frac{\sin\left(\pi\left(\phi_{\max}/2\pi - m\right)\right)}{\pi\left(\phi_{\max}/2\pi - m\right)}\right]^2 \tag{5}$$

It can be seen that the first-order diffraction efficiency reaches 100% when $\phi_{\max} = 2\pi$. When N phase levels are used to discretize the continuous phase profile with a subperiod width of T/N, the resulting phase profile is the subtraction of a sampling phase profile (Supplementary Fig. 5b) from

the original continuous phase profile, as shown in Supplementary Fig. 5c. The overall diffraction efficiency becomes the efficiency of the continuous phase profile multiplied by the zero-order efficiency of the sampling phase profile:

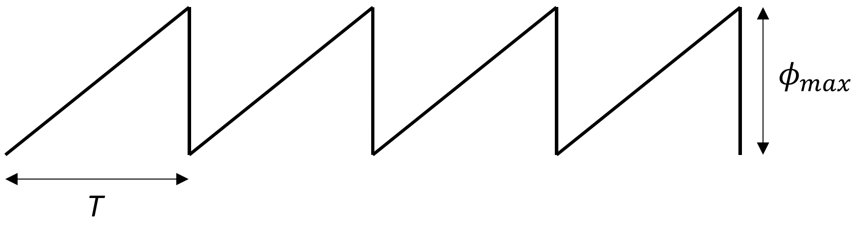

(a)

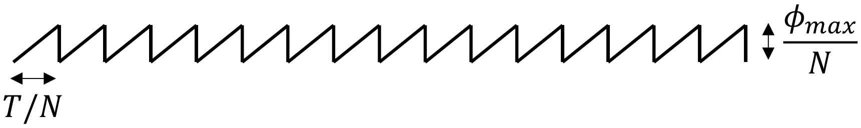

(b)

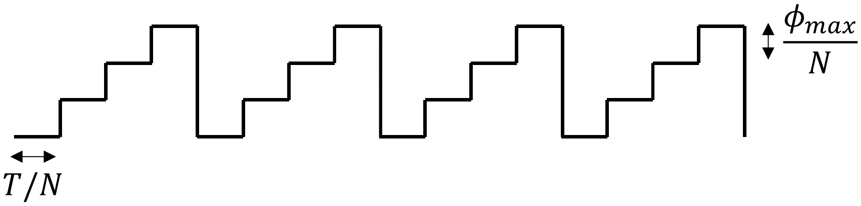

(c)

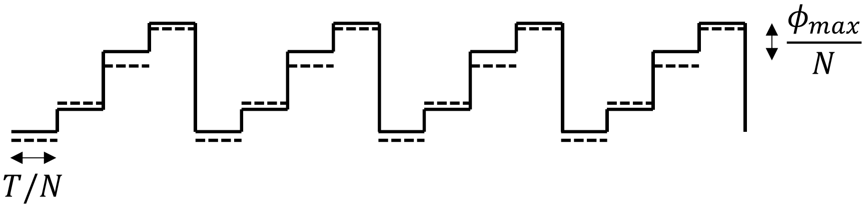

(d)

Supplementary Fig. 5. (a) A continuous phase profile; (b) a sampling phase profile; (c) a discretized phase profile with evenly spaced phase levels; (d) a discretized phase profile with phase errors.

$$\eta_m^N = \left[\frac{\sin\left(\pi\left(\phi_{\max}/2\pi - m\right)\right)}{\pi\left(\phi_{\max}/2\pi - m\right)}\right]^2 \left[\frac{\sin\left(\pi\left(\phi_{\max}/2\pi N\right)\right)}{\pi\left(\phi_{\max}/2\pi N\right)}\right]^2 . \quad (6)$$

When the N phase levels evenly divide the full phase range $\phi_{\max}$, the average phase error, defined as $\Delta\phi_{avg} = \left\langle \left|\phi_{meta} - \phi_{target}\right| \right\rangle$ across a subperiod is:

$$\Delta\phi_{\arg} = \frac{\phi_{\max}}{4N}\,. \tag{7}$$

For example, when $\phi_{\max}$ = 2π and N = 2, 4, and 8, the first-order diffraction efficiency is approximately 41%, 81%, and 95%, respectively. When additional small phase errors are added to each of the meta-atoms (Supplementary Fig. 5d), the first-order diffraction efficiency can be approximated as:

$$\eta_1 = \left[\frac{\sin\left(2\left\langle\left|\phi_{meta} - \phi_{target}\right|\right\rangle\right)}{2\left\langle\left|\phi_{meta} - \phi_{target}\right|\right\rangle}\right]^2. \tag{8}$$

Denoting the average transmittance of the meta-atoms as T_{avg}, an overall FOV for meta-atom selection can be defined as:

$$FOM = T_{avg}\left[\frac{\sin\left(2\left\langle\left|\phi_{meta} - \phi_{target}\right|\right\rangle\right)}{2\left\langle\left|\phi_{meta} - \phi_{target}\right|\right\rangle}\right]^2. \tag{9}$$

Supplementary Note 4 – Comparison to the state-of-the-art

We have compared our varifocal metalens to the other demonstrated non-mechanically active metasurfaces. The Supplementary Table 3 summirizes several examples of the state-of-the-art devices based on various non-mechanical switching mechanisms and highlights their key features which are necessary to enable high-performance reconfigurable optical functionalities.

Supplementary Table 3. Non-mechanically actuated reconfigurable metasurfaces: lenses and deflectors.

	switching mechanism	phase tuning range, °	efficiency, %	diffraction-limited focusing	numerical aperture	contrast ratio, dB
This work (lens)	phase change in GSST	360	23/21	Y	0.45/0.35	29.5
Yin, et al[3] (cylindrical lens)	phase change in GST	360	5/10	N	0.52/0.29	N/A
Shirmanesh, et al [4] (deflector)	ITO electro-optical modulation	270	7-19 (bulk reflectance)	N/A	N/A	N/A
Hu, et al[5] (lens)	nanoembossing of top polymer layer	90	<10 (simulation)	N	0.47/0.82	N/A
Wu, et al[6] (deflector)	electro-optic effect in MQWs	65	<7	N/A	0.12/0.19	N/A
Kim, et al[7] (deflector)	metal-insulator transition in VO_2	250	<5 (bulk reflectance)	N/A	N/A	N/A
Li et al[8] (deflector)	reorientation of liquid crystal molecules by external electric field	240	36/26	N/A	0.2/0.2	15

Supplementary Note 5 – Metalens' bandwidth

To study the operational bandwidth, we performed additional simulations of the meta-atom responses (phase and amplitude) over a range of wavelengths and calculated the corresponding Strehl ratios of the reconfigurable metasurface using the diffraction integral model. As shown in Supplementary Fig. 6, the results show that the diffraction-limited bandwidth (Strehl ratio > 0.8) of our metalens is about 80 nm and 100 nm for amorphous and crystalline states, respectively. This is in a good agreement with the dispersion behavior of an ideal flat lens designed with the same center wavelength (5.2 μm) but without the wavelength-dependent phase/amplitude variance at the meta-atom level.

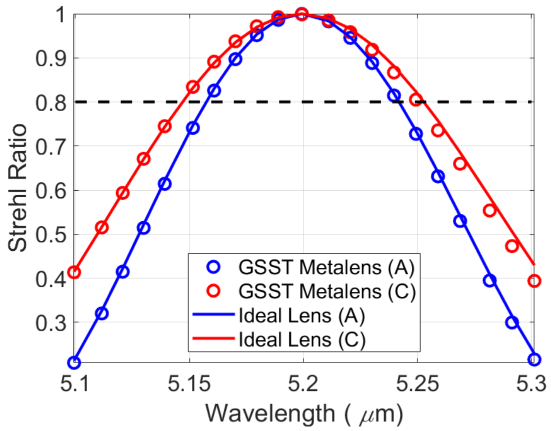

Supplementary Fig. 6. Simulated Strehl ratio of the GSST metalens compared to an ideal lens designed at the same center wavelength (5.2 μm) The wavelength range spans from 5.1 to 5.3 μm (corresponding to frequencies of 56.6 and 58.83 THz).

Here we provide more details on the wavelength-dependent responses of our meta-atoms. In principle, the designed meta-atoms can be considered as Huygens' sources, and each individual meta-atom is represented by the combination of electric and magnetic dipoles that follow a Lorentzian frequency dependence with different resonant positions. The phase shift tuning at the specific state (amorphous or crystalline) is achieved by engineering meta-atom shapes, while the phase shift tuning between different states (amorphous to crystalline or inversely) is realized by varying dipole resonant frequencies with respect to various material indices. This indicates the narrow-band nature of the Huygens-type metasurfaces. As shown in Supplementary Fig. 7, we simulated the amplitude and phase responses of the 16 designed meta-atoms over a wide spectrum (4 μm to 7.5 μm), then calculated average amplitudes for the 16 meta-atoms (Supplementary Fig. 7a). Afterwards, we evaluated the average phase differences (in degrees) at each frequency point comparing to the "standard" phase shift (2π evenly divided by 4 levels) at working frequency of 5.2 μm (57.7 THz). The average phase errors are shown in Supplementary Fig. 7b. As expected, the phase errors increase when operating frequency deviates from the designed working frequency.

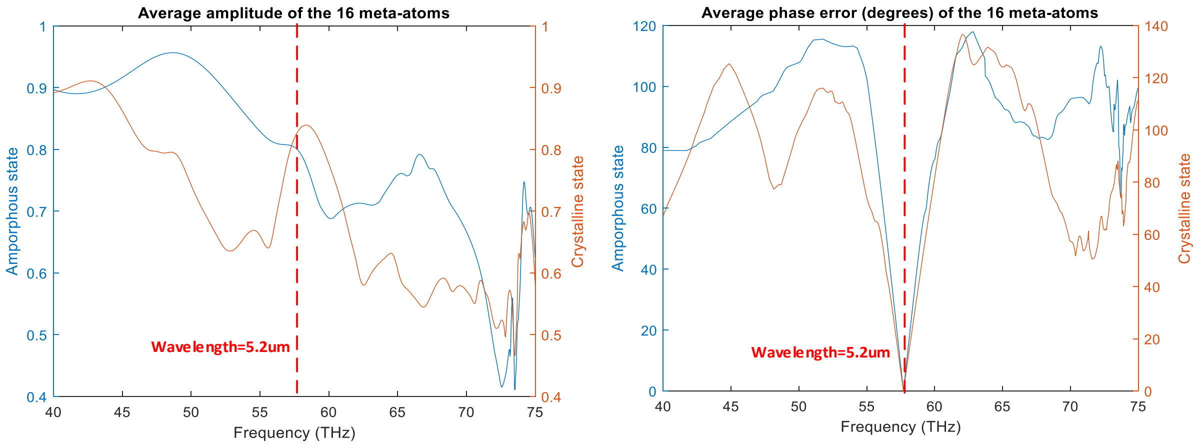

Supplementary Fig. 7. (a) Average amplitudes of the 16 meta-atoms in the 40 to 75 THz frequency range. (b) Average phase errors of the 16 meta-atoms with respect to the "standard" phase shift at working frequency. The results for amorphous state are shown in blue color, while the results for crystalline state are shown in red. The working frequency (57.7 THz, 5.2 μm) is marked with a red dashed line.

Supplementary Note 6 – Polarization-insensitive reconfigurable meta-atoms

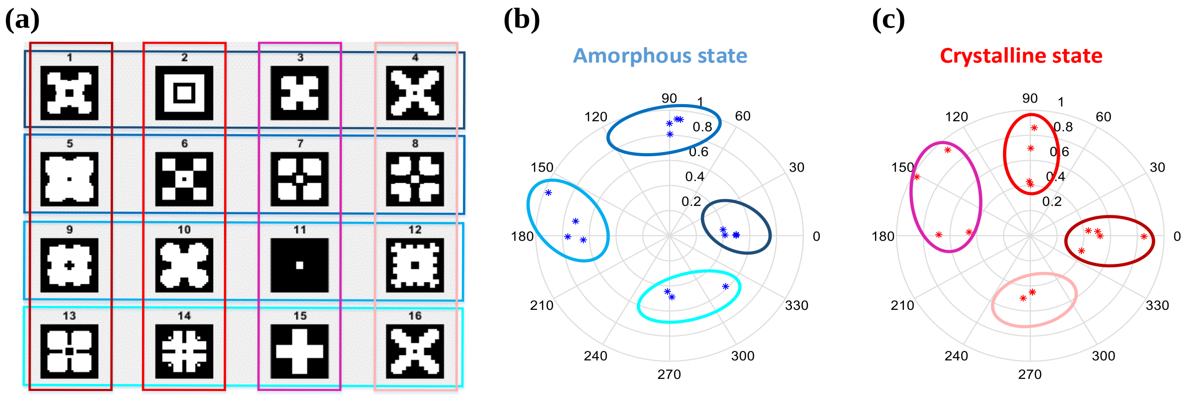

Supplementary Fig. 8. (a) Schematic top-view of the 4-fold symmetry 2-bit meta-atom designs; (b) simulated phase and amplitude of the 16 meta-atoms in amorphous state; (c) simulated phase and amplitude of the 16 meta-atoms in crystalline state.

The most straightforward approach to design meta-atoms with a polarization-independent performance is to find the unit-cell cross-section geometries with 4-fold symmetry. As an example, we selected 16 meta-atom designs with randomly-generated 4-fold symmetrical shapes (Supplementary Fig. 8). The selected meta-atoms can provide full 2π phase coverage and enable the required 16 phase-delay responses in amorphous and crystalline states. Similar to the original designs shown in Fig. 2k of the main manuscript, this meta-atom set can be employed to generate arbitrary wavefronts in both states. The simulated values of phase shifts and transmittance of the polarization-independent meta-atoms are listed in Supplementary Table 4. The average transmitted field amplitudes of 4-fold symmetric patterns are 0.65 and 0.6 in A-state and C-state, respectively, which can be further improved by increasing design degrees of freedom and using advanced optimization methods[9].

Supplementary Table 4. Phase delays and transmittances of the polarization-independent meta-atoms in amorphous and crystalline states of GSST.

cell	amorphous		crystalline	
	phase, °	T, %	phase, °	T, %
1	6.9	16.21	3.8	25.43
2	0.1	24.71	89.7	48.60
3	0.7	17.14	179.3	46.76
4	1.0	24.78	271.9	20.21
5	89.7	65.41	359.5	72.20
6	90.0	79.98	89.6	16.61
7	86.6	86.73	176.4	21.01
8	84.9	86.39	263.9	25.04
9	180.6	57.92	359.7	27.21
10	182.9	41.67	91.0	18.69
11	159.2	93.76	151.1	93.99
12	170.6	50.63	342.5	16.06
13	267.9	19.80	5.2	18.96
14	272.0	23.92	88.0	74.01
15	316.0	33.82	132.1	84.77
16	361.0	24.78	271.9	20.21

Supplementary References

1. Swanson, G. J. Binary Optics Technology : The Theory and Design of Multi-level Diffractive Optical Elements. *Contract* 1–53 (1989).

2. Lee, W. H. Computer-generated holograms: Techniques and applications. *Prog. Opt.* **16**, 119–232 (1978).

3. Yin, X. *et al.* Beam switching and bifocal zoom lensing using active plasmonic metasurfaces. *Light Sci. Appl.* **6**, e17016 (2017).

4. Shirmanesh, G. K., Sokhoyan, R., Wu, P. C. & Atwater, H. A. Electro-optically Tunable Multifunctional Metasurfaces. *ACS Nano* **14**, 6912–6920 (2020).

5. Hu, J. *et al.* Lattice-Resonance Metalenses for Fully Reconfigurable Imaging. *ACS Nano* **13**, 4613–4620 (2019).

6. Wu, P. C. *et al.* Dynamic beam steering with all-dielectric electro-optic III–V multiple-quantum-well metasurfaces. *Nat. Commun.* **10**, 1–9 (2019).

7. Kim, Y. *et al.* Phase Modulation with Electrically Tunable Vanadium Dioxide Phase-Change Metasurfaces. *Nano Lett.* **19**, 3961–3968 (2019).

8. Li, S. Q. *et al.* Phase-only transmissive spatial light modulator based on tunable dielectric metasurface. *Science* **364**, 1087–1090 (2019).

9. An, S. *et al.* A Deep Learning Approach for Objective-Driven All-Dielectric Metasurface Design. *ACS Photonics* **6**, 3196–3207 (2019).
